# A Customized Intervention Program Aiming to Improve Healthy Eating and Physical Activity Among Preschool Children: Protocol for a Randomized Controlled Trial (Iran Healthy Start Study)

**DOI:** 10.2196/11329

**Published:** 2018-12-21

**Authors:** Atieh Mehdizadeh, Mohsen Nematy, Majid Khadem-Rezaiyan, Majid Ghayour-Mobarhan, Mohammad Ali Sardar, Anne Leis, Louise Humbert, Mathieu Bélanger, Hassan Vatanparast

**Affiliations:** 1 School of Medicine Mashhad University of Medical Sciences Mashhad Islamic Republic of Iran; 2 College of Medicine University of Saskatchewan Saskatoon, SK Canada; 3 College of Kinesiology University of Saskatchewan Saskatoon, SK Canada; 4 Department of Family Medicine Université de Sherbrooke Moncton, NB Canada; 5 College of Pharmacy and Nutrition University of Saskatchewan Saskatoon, SK Canada

**Keywords:** childhood obesity, healthy eating, Healthy Start/Depart Sante, Iran, intervention, parent, physical activity

## Abstract

**Background:**

Prevention of childhood obesity is a key approach to the primary prevention of noncommunicable diseases. Several models, based on the population health approach and aligned with ecological models, are used to design childhood obesity prevention programs around the world.

**Objective:**

This study aims to introduce the design and evaluation plan of “Iran Healthy Start (IHS)/Aghazi Salem, Koodake Irani”—the customized Iranian version of Canadian Healthy Start/Départ Santé health promotion program—which is now being developed in Mashhad University of Medical Sciences (Mashhad, Iran) and focuses on improving physical activity and healthy eating among preschool children.

**Methods:**

We will evaluate the intervention using a pilot randomized controlled design. The components of intervention include customized Decoda Web-based resources for children, an implementation guide for educators and managers, training and monitoring, communication and knowledge exchange, building partnership, and parent engagement. Outcomes include changes in anthropometry, physical activity level, nutritional risk status and dietary intake, and quality of life.

**Results:**

The project is funded by Mashhad University of Medical Sciences. The intervention was completed by the end of March 2018, and the analysis is currently under way. The first results of the IHS intervention program are expected to be submitted for publication in December 2018.

**Conclusions:**

The double burden of malnutrition in early years children is a major health concern in developing countries. This justifies the need for health promotion programs that are specifically designed to target both overnutrition and undernutrition prevention. If the efficacy approved, the IHS could potentially be a comprehensive health promotion program for young children whose lifestyle behaviors can be improved toward a healthy future life in a nutrition transition setting.

**Trial Registration:**

International Clinical Trials Registry Platform IRCT2016041927475N1; https://en.irct.ir/trial/22497

**International Registered Report Identifier (IRRID):**

RR1-10.2196/11329

## Introduction

At present, noncommunicable diseases (NCDs) and obesity—as the main predisposing factor of NCDs—are the most common cause of mortality and morbidity, with a higher prevalence in low-income and middle-income countries [1]. The World Health Organization (WHO) has introduced NCDs as the major challenge for development and emphasizes the need for urgent action to prevent and control NCDs [2]. Among the NCDs’ risk factors, obesity needs particular attention, as metabolic syndrome and diabetes are directly associated with obesity [3,4]. Early childhood obesity is associated with a higher risk of developing NCDs at a younger age and premature death in adulthood [5,6], as adiposity tracks into adulthood [7]. Prevention of childhood obesity is a key approach to the primary prevention of NCDs [3]. Based on the WHO report, in 2016, an estimated 42 million children aged <5 years are overweight or obese, while around 70% of them live in Asia and Africa [8]. In Iran, according to the latest report of an Iranian national survey on May 2015, which was conducted in 30 provinces, the prevalence of overweight, obesity, and abdominal obesity among children and adolescents aged 6-18 years was 9.7%, 11.9%, and 19.1%, respectively [9].

After the announcement of the WHO on February 2015, regarding the urgency of ending childhood obesity around the world, societies and governments took urgent and meaningful action to address this issue. Childhood obesity is a complex and multidimensional health problem, and usually, single and narrow target interventions do not succeed. Evidence suggests that improving healthy eating and physical activity behaviors are the cornerstone for weight management strategies [10,11], along with decreasing sedentary activities [12,13] and maintaining healthy sleep patterns [14,15], Most recent intervention strategies have focused on modifying environment in a way that provides healthy options for children and increases opportunities for physical activity and healthy eating.

In developing countries, such as Iran, nutrition transition is occurring side-by-side with epidemiological transition. A history of low birth weight or stunting, which still exists in developing countries, is a risk factor for later overweight and obesity in children and consequent cardiovascular diseases or diabetes [16]. According to the data released from the WHO, United Nations International Children’s Emergency Fund, and World Bank Group in 2016, Middle East and North Africa, which mainly include higher middle-income countries, have the highest prevalence of overweight and obesity (10.7%) after Eastern Europe and Central Asia (12.8%) among global regions, while the prevalence of coexisting stunting is 15.3% among Middle Eastern and North African countries [17]. The presence of both overweight and underweight in a population or even in families is recognized as “double burden of malnutrition” and highlights the importance of nutrition in early life and its close relation with later-life health conditions [8]. In such a situation, the question arises, “How can an obesity prevention program be conducted, where the problem of overweight and underweight simultaneously exists among children?”

Several childhood obesity prevention programs using different models based on the population health approach [18,19], and aligned with ecological models [20], have been designed and conducted around the world with different scientific rationale [21,22]. Interventions that adhere to principles of the population health approach are designed in accordance with the fact that in a comprehensive population health approach, all levels of impact, such as the intrapersonal (psychological and biological), interpersonal (psychological and social), institutional, community (resources and facilities), and governmental policy, should be included and stimulated to promote the achievements. Healthy Start/Départ Santé (HSDS) is a health promotion program, designed as a population health intervention aiming at promoting physical activity and healthy eating among both Anglophone and Francophone preschoolers in early daycare or preschools in Canada. HSDS is composed of the following 6 interlinked components: (1) evidence-based resource, “Literacy, Education, Activity, Play (LEAP)” from the Decoda literacy Web-based resources [23,24]; (2) HSDS implementation guide; (3) training and monitoring; (4) intersectoral partnership; (5) additional resources; and (6) communication, knowledge development, and exchange. In the evaluation of the HSDS intervention program, 230 children aged 2.5-4 years from very diverse sociocultural and economic backgrounds, from 10 childcare centers, were involved in the project. The results clearly represented groundbreaking and innovative work. Improvements were seen in healthy eating children, physical activity, and childcare environment. Healthy Start has been developed and initiated in the Saskatchewan province of Canada and is expected to expand to the whole country and other countries around the world. The first report of HSDS was released on November 2012 [25].

To address the challenge of childhood obesity and the double burden of malnutrition in a developing country, we decided to calibrate and customize HSDS as a health promotion initiative, which is now successfully running in 2 provinces in Canada [26]. “Iran Healthy Start (IHS)/Aghazi Salem, Koodake Irani” is the customized Iranian version of Canadian HSDS health promotion program, which is now being developed in Mashhad University of Medical Sciences (MUMS; Mashhad, Iran) and focuses on improving physical activity and healthy eating among preschool children. The principles of IHS are adapted from the original Canadian HSDS, considering unique conditions that exist in the Iranian culture, preschool education bylaws, curriculum, and environment.

**Study objectives.**

1. To customize and implement the health promotion program (Iran Healthy Start), aligned with preschool bylaws in Iran
2. To determine whether Iran Healthy Start program can:
  - Increase physical activity level and attraction to physical activity among preschoolers
  - Reduce sedentary behaviors at home among preschoolers
  - Improve anthropometric parameters in preschoolers toward healthy weights
  - Improve quality of life in preschoolers
  - Improve eating habits and nutrition risk among preschoolers
3. To evaluate the feasibility, attrition rate, as well as facilitators and barriers for implementing this program in Iranian preschools
4. To calibrate measurement tools: validating the Persian translation of Nutrition Screening Tool for Every Preschooler and Children Attraction toward Physical Activity

From our point of view, some characteristics of HSDS make it more relevant to what we need. The key characteristic of HSDS is that it is based on evidence-based ecological framework [27] and adheres to the population health approach. Furthermore, it introduces strategies for all levels of influence. Another advantage of this program is its multicultural nature, which makes the program more relevant at the international stage and makes the customization easier, although it requires fundamental modifications to be feasible for Iranian preschool children. HSDS targets all children, regardless of their weight or risk of obesity, which is the primary prevention initiative (health promotion) in the public health approach. This level of prevention includes fundamental activities that are directed at reducing the risk of exposure to a risk factor in an individual or the population [28].

Textbox 1 shows the objectives of this study.

## Methods

### Study Description

This section is addressed on the basis of the Standard Protocol Items: Recommendations for Interventional Trials [29]. In a multisectoral approach, the key stakeholders, including Departments of Nutrition, Chancellor for Health and Chancellor for Research in MUMS, Provincial Education Department, and Nutrition Affairs Department of the Iranian Ministry of Health and Medical Education, are involved in the “IHS” program. The IHS is supported and funded by MUMS. A memorandum of understanding was signed between MUMS and HSDS lead organization (Saskatchewan Network for Health Services in French—Réseau de Santé en Français de la Saskatchewan) in Canada.

### Providing the Intervention Material

Prior to the evaluation, we need to provide the material and equipment for children and educators, as well as materials for engaging parents. This step requires the active involvement of the expert panel for the development and customization of the educational content and assistants for providing the toolkit and supplementary materials for implementation.

#### Development of the Intervention Components, Materials, and Customization Process

Considering some basic differences between Iranian and Canadian education environment and principles, the following criteria were determined for the development and customization of the intervention components and content: (1) Iranian preschool education bylaws; (2) current nutrition and physical activity guidelines for preschoolers; (3) physical area or environment in most preschools in Iran; (4) Iranian common plays, lyrics or songs, and play tools among preschool children; (5) Iranian culture (menu, common foods, and eating culture); (6) known foods preferred by Iranian children of this age; and (7) commonly available foods and their cost. For this purpose, an expert panel, including a nutritionist, physical activity expert, epidemiologist, representative of Provincial Education Department, graphist, psychologist, and an expert in children plays and lyrics, gathered in eight, 2-hour meetings to reach a consensus. Accepted modifications for each component or material or activity unit were registered by the secretary.

#### The Intervention

The IHS program has 6 components (Table 1):

1. *Customized LEAP*: Comprises two illustrated handbooks.
  - Physical activity: containing 20 activity units along with a complementary chapter containing information for educators (ISBN: 2-25-7457-600-978).
  - Nutrition: containing 20 activity units along with a complementary chapter containing information for educators (ISBN: 9-26-7457-600-978).
  The activity units were translated and customized on the basis of the criteria discussed during the “customization” process. This handbook is accompanied with “Healthy-kid Toolkit,” which contains utilities and materials for LEAP activity units.
2. *IHS Implementation Guide*: A handbook for managers and educators containing modified self-assessment tool, as well as principles of the program, action planning, policies and practices, details of implementation, log pages, and report pages for both educators and managers. It also contains a suggested healthy weekly menu for serving a snack or a hot meal for children at preschool.
3. *Training and Monitoring*: Comprises on-site training workshop followed by a supplementary booster session during the implementation (3-4 hours), as well as ongoing support through mobile and internet-based contacts (telegram) and weekly visits. The initial workshop introduces the program, objectives, customized LEAP activity units, IHS Toolkit, and resources. Educators and managers of intervention centers are invited to become a member of the IHS telegram channel, which is the most popular internet-based social network among Iranians. The implementation is monitored by weekly visits and daily logs and checking videos and photos while children do the activities every day.
4. *Building Partnership*: Attracting the participation of key stakeholders and policy makers:
  - Ministry of Health and Medical Education: Nutrition Affairs Department of the Ministry was informed about the details of the project from the inception of the project. The results of this pilot study will be reported to the aforementioned department for further steps toward the national implementation of the program.
  - Provincial broadcasting (radio and television): A television show and radio talks related to childhood obesity, physical activity in children, healthy nutrition, parenting, and many other related topics were recorded to introduce the program and report the future outcomes.
  - Provincial Education Department: Aiming at incorporating this program or any of its components into the current preschool education bylaws, several meetings were arranged with heads-in-charge.
5. *Parent Engagement*: This is the main distinction between HSDS and IHS and includes the following:
  - A scientific, user-friendly, and simple book entitled, “Knowledgeable Parents, Healthier Children,” for parents aiming at improving their knowledge and practice regarding healthy eating for the whole family, especially their preschool child (ISBN: 5-24-7457-600-978).
  - Routine monthly meetings between parents and IHS team nutritionists.
  A 107-page book, which is written for parents, contains 5 main chapters as follows: (1) childhood obesity, the epidemic health problem; (2) the importance of healthy nutrition and physical activity for children; (3) the role of parents in children weight management; (4) parental challenges and worriment; and (5) specific nutrition and activity considerations for a preschool child. Parents are asked to read each chapter during one month. In monthly meetings, the content of each chapter is again explained and discussed for parents. These parent-nutritionist meetings are mutual, and parents are allowed to ask questions or share their comments or experiences regarding children’s nutrition and activity issues. This book is written by 2 team members (MN and AM). Before releasing this resource, 3 collaborating mothers were selected to read the book and share their comments regarding the fluency, practicality, and usefulness of content, as well as suggestions for adding topics or other required data.
6. *Communication and Knowledge Exchange*: The purpose of this component is maintained by a comprehensive website [30] containing details of the program, pages for managers, educators, parents, and children, as well as social media tools and reports of the study findings for the stakeholders. This component is expected to improve knowledge, engagement, motivation, and level of cooperation in stakeholders, as well as knowledge translation and dissemination of the program and findings.

Tables 1 and 2 show a brief introduction of components of the Canadian HSDS and IHS programs. The Canadian HSDS program [26] consisted of children aged 2-5 years, and the duration of intervention was 11 months. The Iranian IHS program consisted of children aged 4-6 years, and the duration of intervention was 6 months.

**Table 1:** Brief introduction of the components of the Healthy Start/Départ Santé program.

Program components	Implementation items
Literacy, Education, Activity, Play	Illustrated handbook containing healthy opportunities for preschoolers and Food Flair
Healthy Start/Départ Santé Implementation Guide	Handbook for directors and educators
Training and monitoring	On-site training session, which includes the monitoring of Healthy Start/Départ Santé in the center, as well as a supplementary training session (1-2 hours)
Partnership	Partnerships and linkages with key stakeholders, decision, and policy makers
Additional resources	Supplementary resources from the government
Communication, knowledge development, and exchange	Hands-on material, website, social media tools, dissemination of evaluation findings, and communications through mainstream media releases

**Table 2:** Brief introduction of the components of the Iran Healthy Start program.

Program components	Implementation items
Customized Literacy, Education, Activity, Play	Illustrated handbook containing customized Literacy, Education, Activity, Play based on the Iranian preschool education bylaws, current nutrition, and physical activity guidelines for preschoolers, physical area, or environment in most preschools in Iran, Iranian common plays and lyrics or songs among preschool children, Iranian common playing tools and materials, Iranian culture (menu, common foods, and eating culture), known foods preferred by Iranian children of this age, and commonly available foods and their cost.
Iran Healthy Start implementation guide	Translated handbook for directors and educators
Training and monitoring	On-site training workshop and a supplementary booster session. The implementation is monitored by weekly visits and daily logs and sending videos and photos while children do the activities
Building partnership	Attracting the participation of the following: Ministry of Health by reporting results of the pilot study and proposing the national governmental program Iran broadcasting (radio and television) to introduce the program and report the outcomes Provincial Education Department for incorporating the designed material into the preschool education bylaws
Parent engagement	A 107-page book provided for parents, containing 5 main chapters to be discussed in each gathering session of parents and IHS team nutritionist (routinely every month)
Communication and knowledge exchange	Website, social media tools, and reports of the study findings for the stakeholders

### Development of the Evaluation Plan and Designing the Pilot Study

#### Obtaining Required Approval and Licenses

Conducting any educational interventions in preschools and schools in Iran needs a valid license from the Provincial Education Department. This license was obtained on October 8, 2016. MUMS and Chancellor for research provided the financial support. The study is approved by the Ethics Committee of MUMS (code: IR.MUMS.fm.REC.1395.208; September 25, 2016) and registered in Iranian Registry for Clinical Trials (ID: IRCT2016041927475N1; November 12, 2016) and is accessible through the WHO database of clinical trial registries. A comprehensive memorandum of understanding was entered between Chancellor for Health, MUMS and Saskatchewan Network for Health Services in French—Réseau de Santé en Français de la Saskatchewan (April 2017).

#### Development of the Evaluation Plan

##### Study Design

**Figure 1:**
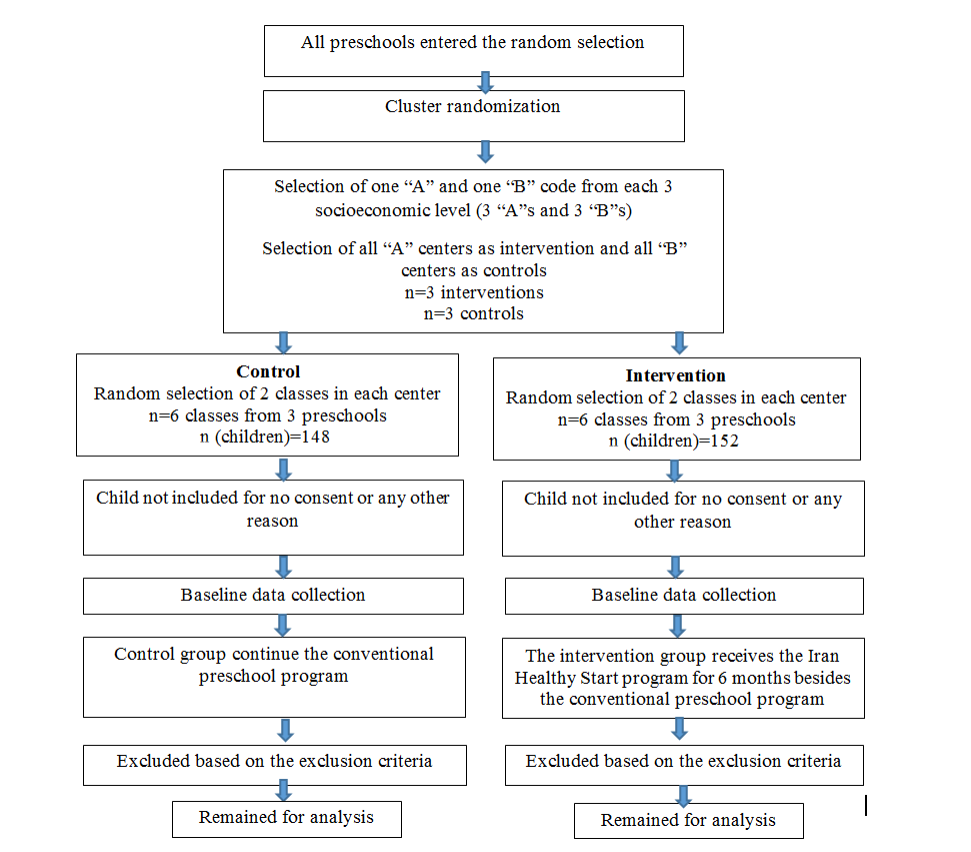
Flow diagram of the recruitment process.

The evaluation of the IHS program is designed as a pilot randomized controlled trial. For recruitment, officially registered preschools in the Provincial Education Department database are stratified according to the socioeconomic status of people living in that area. The three levels of high, middle, and low socioeconomic areas are defined according to the categorization of the Provincial Education Department. Two preschools are randomly selected from each socioeconomic level (a total of 6 centers) and then allocated to the intervention and control groups (Figure 1). Overall, 3 centers in the intervention group (each center belongs to a different socioeconomic level) and 3 centers in the control group are defined. The selected centers are contacted in person and provided with comprehensive information. After confirmation of managers, 2 classes are randomly selected for enrollment (6 classes in the intervention group and 6 classes in the control group). Then, the parents of eligible children are invited in a meeting in preschool, and informed consent is obtained after a brief introduction on the whole program and the role of parents (for parents of the intervention group) and informing parents for data collection and cooperation in filling questionnaires (for parents of the control group). The components of the intervention program for managers, educators, children, and parents are conducted in intervention classes, and control classes receive the conventional preschool education program. Aiming to acknowledge the cooperation of control preschools, the wait-list approach is suggested to the managers to receive the intervention in the next educational year, as well as a comprehensive report for growth, nutrition, and physical activity status of each child, which is given after the intervention, is completed.

The intervention lasts for 6 months. The duration of an educational year in preschools in Iran is the same as elementary schools and lasts for 8 months (October-May). From March 21, there are around 2-week New Year (Norouz) holidays. The intervention will be completed before Norouz holidays. The required data are collected at baseline and after the completion of the intervention period.

##### Study Setting

Mashhad is a large city in the northeast of Iran and has 7 areas, based on the classification of the Provincial Education Department. Preschools in Mashhad are nonprofit and are under the supervision of the Provincial Education Department. Most of the preschools in Mashhad start at 7:30 am and close at 2:00 pm. This is the official time, but most children, even those whose parents are employed, come at 8:30-9:00 am and leave the preschool at 12:00-12:30 pm. In addition, preschool service taxis follow this time (8:30-12:30). Therefore, children spend around 4 hours at preschools.

##### Participants and Data Collection

In the Iranian preschool bylaws, preschool is not a mandatory level, and all children aged 4-6 years can be registered either in preschool-1 (age, 4-5 years) or preschool-2 (age, 5-6 years). All children aged 4-6 years of the selected classes of each center are included in the study after a valid consent from their parents or legal guardian, while those with known chronic disease, such as cardiovascular, respiratory, endocrine, or musculoskeletal problems—after physician inquiry—as well as those who need to adhere to a specific diet, such as gluten-free or phenylalanine-free diets, are excluded.

Data reflecting the nutrition status, diet quality, physical activity and sedentary behaviors, quality of life, and anthropometry of children, as well as the general sociodemographic status of participants and their families are collected at baseline and after the intervention is completed (Table 3). Data collection is performed by a team of 4 nutritionists who are given comprehensive training to standardize the data collection. They learned how to consistently obtain anthropometric measurements, fill the required questionnaires, and train the use of pedometers and filling the parent-report questionnaires to parents. Data entry is done by one person for consistency (a PhD student). Each preschool takes about 7-10 days to collect all the required data. As parents’ cooperation is very important in the data collection, aiming to acknowledge their cooperation, especially in control preschools, a gift for children and a free nutrition consultation for children and their parents, as well as a comprehensive report for child growth, nutrition, and activity status, are dedicated.

As this is a pilot study and we cannot estimate the attrition rate, there is no need to define the sample size, but sample characteristics represent the population of preschool children in Mashhad.

**Table 3:** Overview of the study process.

Group and action		Months	
**Intervention**			
	Introduction to managers		1
	Training educators (workshop)		1
	Introduction to parents		1
	Baseline data collection		1
	LEAP activity units every day		1-6
	Providing ongoing support for educators		1-6
	Booster session for educators at month 3		1-6
	Resources and monthly sessions for parents		1-6
	Filling daily logs, weekly reports and comments (increasing intervention efficacy)		1-6
	Postintervention data collection		6
	Feedback from staff and parents		6
**Control**			
	Baseline data collection		1
	Conventional programs in preschools		1-6
	Final data collection		6

**Table 4:** A list of variables, tools, and data collection points.

Variable	Assessment tool	Timing
Sociodemographic data	Questionnaire	Baseline
Anthropometry (children)	Weight, height, waist and arm circumference, body mass index percentile, and body mass index *z*-scores for age	Baseline/End
Nutrition risk (children)	Nutrition Screening Tool for Every Preschooler	Baseline/End
Food intake and eating habits	24-hour recall×3	Baseline/End
Physical activity level (children)	Children attitude toward physical activity Physical activity level at home Physical activity level by pedometers
Quality of life (children)	Pediatric Quality of Life Inventory questionnaire	Baseline/End
Efficiency and adequacy of the parent material	Interview with parents	End
Feasibility of the program (weaknesses and strengths)	Interview with managers and educators	End

#### Variables of Interest

Several variables are considered to assess the defined objectives, which are specified earlier. Table 4 presents the main outcome measures that are administered among both intervention and control participants.

##### Sociodemographic Information

This questionnaire provides demographic data, such as parents’ status, age, education level, job, family income, as well as some known childhood obesity associated variables such as parent-reported weight and height, duration of child exclusive and nonexclusive breastfeeding, age of solid food introduction, age of starting kindergarten, eating in front of screen, physical and sedentary behaviors, and some general parenting habits such as child force-feeding, use of food as reward or punishment, parents role model or the presence at meal time, and parental controlling approach.

##### Nutritional Assessment

###### Diet Quality

Children’s food intake is assessed by the 24-hour recall for each child, which is filled for 3 days (2 regular days and 1 holiday) with a 14-day interval, at baseline and after the intervention. As parents provide children’s midday snack themselves and educators are obligated to provide daily reports for parents about the amount of snack their child has eaten, parents are the best source for receiving the data of children’s food intake. The Youth Healthy Eating Index (YHEI) was used for the analysis of the diet quality out of food recalls [31,32]. The YHEI focuses on the total fat, sodium, and saturated fat in children’s diet by examining food choices rather than a direct calculation of the nutrient intake. Furthermore, the YHEI focuses on trans-fatty acids, added sugar, and low fiber in children’s diet, unlike the conventional Healthy Eating Index [31].

###### Nutrition Risk

The NutriSTEP [33,34] has been first designed and validated in Canada in different languages for assessing eating habits and nutrition problems in children aged 3-5 years. A license was obtained from the developers of NutriSTEP questionnaires. The Persian version of NutriSTEP was used after translation and cross-cultural adaptation [35]. The NutriSTEP scoring system categorizes children into low, medium, and high risk in terms of nutritional status. It covers 4 main domains related to children’s food intake as follows: (1) food and fruit intake; (2) physical growth and development; (3) factors affecting the food intake and eating behavior; (4) physical activity and sedentary behavior.

###### Anthropometry

Weight, height, midarm circumference, and waist circumference are obtained from all participants, at baseline and after completion of the intervention, according to the standardized protocol [36]. Weight is measured using the Beurer BG13 Digital Scale, Germany, with a measuring rod of 0.1 kg and height using SECA 206 stadiometer, Germany, with a measuring rod of 0.1 cm. The waist and arm circumference are measured to the nearest 0.1 cm. Body mass index (BMI), BMI percentile, and BMI *z*-score are calculated using AnthroPlus software, version 1.0.4, Geneva, WHO, 2009 [37].

##### Physical Activity Assessment

The physical activity level is assessed using subjective and objective instruments.

###### Children Attitude Toward Physical Activity

This index is assessed through a 25-item, Likert scale validated questionnaire, which is answered by children with the assistance of an interviewer (educators) and scores children’s attitude and degree of attraction toward physical activity [38,39]. The Children Attraction toward Physical Activity (CAPA) original questionnaire was translated into Persian and culturally adapted, based on the current guidelines for translation and cross-cultural adaptation of questionnaires [35]. The Persian version of the CAPA questionnaire has excellent internal consistency among Iranian preschool children after omitting 4 questions (Cronbach alpha=.93).

###### Physical Activity Level (Subjective)

The level of physical activity at home is assessed by a validated parent-reported questionnaire [40], after translation into Persian and cross-cultural adaptation [35].

###### Physical Activity Level (Objective)

Pedometers are used for this purpose. Cost, memory capacity, and use burden for children (weight, size, probability of child manipulation, and falling), as well as accuracy and validity, are considered for the selection of pedometers, based on McClain and Tudor-Locke’s suggestion [41]. For this purpose, we use Omron HJ­320 Tri­axis pedometer at baseline and after the intervention. Further to its reasonable price, Omron HJ­320 has several advantages to be used in children. It locates on child’s waist, which is the body axis and gives a better estimation of the physical activity level. Furthermore, it has a 7-day memory and records the number of steps during 7 consecutive days. It is very light (45 g), easy to both set and use, and impossible for child’s manipulation. All pedometers are set based on children’s average length of steps.

##### Quality of Life

Quality of life measures are increasingly being used as an index of population health status after the constitution of the WHO on the definition of “Health” that is a state of complete physical, mental (including emotional and cognitive), and social well-being [42].

We use the Pediatric Quality of Life Inventory 4.0 questionnaire, for children (age 5-7 years) for this purpose [43,44], which has a previously validated Persian version [45] and contains 23 items encompassing 4 fields of children function—physical, emotional, social, and school—and is answered in a 5-point Likert scale.

##### Qualitative Interview With Parents, Educators, and Managers

We ask educators to write their comments about the feasibility, attraction, useful hints, and suggestions for increasing children’s and parents’ motivation to actively participate in the program, read the resource, and attend the training sessions during the intervention. After the intervention is completed, we have an in-person interview with managers, educators, and a number of parents to figure out some qualitative data regarding the feasibility of the program in Iran preschools education environment, as well as strengths and limitations of the program in components, materials, and implementation aspects. Parents in the intervention group are stratified to 4 strata, based on the portion of the book they were supposed to read during 6 months—(1) <25%; (2) 25%-50%; (3) 50%-75%; and (4) >75%. Then, 10 parents are randomly selected from each stratum and are contacted through a phone call, and qualitative data regarding the fluency, practicality, usefulness, comprehensiveness, and the potential barriers causing a lack of parental cooperation are collected.

##### Data Analysis

Descriptive analysis (frequency, percentage, mean, and SD) will be used to characterize and compare the basic characteristics of participants in the intervention and control groups. Outcomes related to nutrition and physical activity will be compared between the intervention and control groups to seek any possible improvements following the intervention. Considering the number of intervention (n=152) and control (n=148) participants, which anticipates a normal distribution, independent sample *t* test, and chi-square tests will be used for between-group comparisons, while paired sample *t* test and MacNemar tests will be used for analyzing within-group changes. Any possible covariates will be controlled with the analysis of covariance test. All tests are 2-tailed, and a significance level of <.05 will be considered statistically significant. Data analysis will be done by IBM SPSS Statistics for Windows, version 23.0 (IBM Corp).

### Implementation

#### Training Educators

Aiming at empowering educators and integration of the implementation among intervention centers, a workshop at baseline and a booster session at the middle of the intervention period is conducted. Details of LEAP activities, activity demonstrations, role of educators, collaboration with parents, and other related implementation hints are presented and discussed.

#### Process Evaluation and Monitoring

The Reach, Effectiveness, Adoption, Implementation, and Maintenance framework is used to assess the implementation process [24]. Educators are asked to fill logs every day containing the code of activity unit, the time spent on each unit, and feedback of children learning. Photos or videos of LEAP activities are received by the implementation team every day through internet-based contacts (telegram channel). A team member has on-site weekly visits to ensure the standard implementation of the whole program and the activities.

## Results

The project is funded by MUMS. The intervention was completed by the end of March 2018, and the analysis is currently under way. The validation papers of the Persian version of questionnaires (NutriSTEP and CAPA) are now completed and submitted for publication. The first results of the IHS intervention program are expected to be submitted for publication in December 2018.

## Discussion

Developing societies need to have a health promotion program that can overcome the double burden of malnutrition. This requires both overnutrition and undernutrition prevention strategies to be combined and integrated into education and health systems. The IHS program has the potential of a comprehensive health promotion program for young children whose lifestyle behaviors can be modeled and improved toward a healthy future life. The IHS program is developed and customized considering several criteria. Looking at the preschool bylaws in Iran, there is not a systematic rule or program or guideline targeting optimal nutrition or structured physical activity for preschool children. Except for some preschools in the capital city (Tehran), almost all preschools are part-time and do not serve lunch for children. Breakfast and snack are routinely up to the parents, but in some preschools, especially in higher socioeconomic areas, a hot meal is served as breakfast or snack, but not lunch. Regarding the physical environment, usually there are 20-30 children in a preschool class, and the area of classes is usually around 12-15 m^2^, with a small yard where around 10-15 children can safely play. Except for rural preschools, most urban centers do not have adequate space for children to even play or have moderate-to-vigorous physical activities. It is mandatory for preschool educators to teach training books in 2 volumes for preschool children during the educational year, which are developed by the Provincial Education Department and are aligned with the conventional educational objectives in the preschool bylaws, which are briefly titled as follows: (1) training physical and movement skills; (2) training emotional attitude and behaviors; (3) training intellectual skills; (4) training moral and social behaviors; (5) developing fondness for learning the Holly book (Quran); (6) training art and beauty; (7) increasing religious tendency; (8) training national identity; (9) training language skills; (10) promoting health and safety level; and (11) familiarity with the nature and conserving the environment. However, there are not any developed and designed activity units focusing on healthy nutrition and structured physical activity in the bylaws.

There are several main differences between the preschool education systems in Iran and Canada, which account for differences in the Healthy Start program components in the two countries. Instead of “Additional Resources” in HSDS, we included “Parent Engagement.” This component is the main difference and better to say the main strength of the IHS protocol. We engaged parents because based on the recent evidence, parents play a crucial role in children’s weight-control management. This role is comparable or even more important than children themselves [46,47]. Therefore, we organized the IHS program in 6 components as follows: (1) customized LEAP: illustrated handbook containing physical activity and nutrition cards accompanied with healthy-kid toolkit; (2) IHS implementation guide; (3) parent engagement; (4) training and monitoring; (5) communication and knowledge exchange; and (6) building partnership.

The results of this evaluation study might be a scientific foundation for future policies and plans. During the study, we will try to have a look at the feasibility and potential implementation problems and barriers in our country. The main obesity prevention program that is now being conducted in some provinces of Iran is “Ending Childhood Obesity (IRAN-ECHO).” This program is designed and implemented in the framework of the WHO-ECHO program [48]. IRAN-ECHO targets only overweight and obese children of school age. Thus, there is a lack of health promotion program in Iran that targets all children at a younger age where health-related behaviors are more modifiable.

This study has several strengths. First is the randomized controlled design of the study and the stratification, which is based on the socioeconomic level of families as an important independent variable influencing the risk of malnutrition among children. Another strength refers to strategies and plans that attract the active involvement of other stakeholders such as parents, preschool educators, and managers and policy makers.

Some limitations of this study need to be discussed as well. The first refers to a large number of students in each class and limited space; therefore, we had to modify physical activity units to those that can be done at class and do not need too much space. Another limitation is that it is not possible to give a menu for snacks or breakfast to families or preschool managers. The former relates to the fact that we have children from economically disadvantaged families who are unable to provide a healthy snack or breakfast menu every day, and the latter refers to the fact that serving breakfast and snack is not a routine daily program of all preschools in Mashhad. Therefore, we decided to focus on that meal and advise the required modifications. Another limitation refers to the fact that we cannot include a follow-up period to evaluate the sustainability and long-term effects of the program because children of preschool level-2 usually leave the preschool at the end of the education year and it is not feasible to track them in other centers.

As a plan, this evaluation study will guide for the development and improvement of the IHS intervention to be qualified as a comprehensive health promotion program and conducted in the whole country, aiming at the prevention of overweight and obesity among preschool children.

## Appendix

Multimedia Appendix 1 Peer review report 1 (in Farsi).

Multimedia Appendix 2 Peer review report 2 (in Farsi).

## Abbreviations

BMI: body mass index
CAPA: Children Attraction Toward Physical Activity
HSDS: Healthy Start/Départ Santé
IHS: Iran Healthy Start
LEAP: Literacy, Education, Activity, Play
MUMS: Mashhad University of Medical Sciences
NCDs: noncommunicable diseases
NutriSTEP: Nutrition Screening Tool for Every Preschooler
WHO: World Health Organization
YHEI: Youth Healthy Eating Index
