# Supplementary material for: A Customized Intervention Program Aiming to Improve Healthy Eating and Physical Activity Among Preschool Children: Protocol for a Randomized Controlled Trial (Iran Healthy Start Study)
Source: JMIR Res Protoc. 2018 Dec 21;7(12):e11329. doi: 10.2196/11329 (PMC6324517; doi:10.2196/11329)
Supplement: Multimedia Appendix 1 [file resprot_v7i12e11329_app1.pdf]

دانشگاه علوم پزشکی مشهد
معاونت پژوهش و فناوری
فرم ارزیابی طرحهای پژوهشی

**عنوان پروژه: «اجرا و سنجش پایلوت مداخله بومی سازی شده با هدف افزایش فعالیت فیزیکی و تغذیه سالم در میان کودکان پیش دبستانی در ایران**	
شماره: 941586	مجریان طرح: دکتر محسن نعمتی
مدت زمان اجرای طرح (ماه): 26ماه	جمع هزینه طرح: 304300000
مقطع (اگر طرح پیشنهادی پایان نامه است): ....)	
اسناد و ضمایم همراه طرح<br>-<br>-<br>-	

- **در صورتی داوری این طرح را بپذیرید که در حوزه این پژوهش تخصص کافی را داشته و اشتراک یا تضاد منافعی با پژوهشگران این طرح نداشته باشید.**
- **لطفا با استقلال کامل، شفافیت (transparency)، اخلاق و رفتار جوانمردانه (fairness) طرح را داوری نمایید.**
- **داوری شما مبنا اصلی تصمیم گیری نهایی برای پذیرش و رد این پژوهش است لذا با دقت فراوان و توجه به نقاط قوت و ضعف، داوری خود را انجام داده و هرنکته ای که به بهبود پژوهش کمک می کند را ذکر نمایید.**
- **توصیه های خود را به شکلی بنویسید که برای طراحان مفهوم، مفید، اختصاصی و حاوی اطلاعات کافی برای اصلاح پژوهش باشد. از ذکر جملات کلی نظیر "نامناسب است" اجتناب نمایید.**
- **در داوری خود فقط و فقط مسائل علمی را لحاظ نموده و در هر بخش حتما نکات مثبت و منفی را ذکر نمایید.**
- **نظر کلی خود در مورد هر بخش را بطور مستقل از بخش های دیگر منظور نمایید و اشکال یک بخش نباید در ارزیابی شما در بخش های دیگر مجددا لحاظ شود.**

هدف و ضرورت انجام کار

در نگارش نکات مثبت و منفی موارد زیر را در نظر بگیرید.

- ✓ هدف چقدر دقیق و شفاف بیان شده است.
- ✓ مشکل و ضرورت انجام پژوهش چقدر شفاف و قابل راستی آزمایی است و آیا در راستای هدف است.
- ✓ تئوری و شواهد ذکر شده برای حمایت از ضرورت انجام کار و مشکل مطرح شده کافی است.
- ✓ هدف و یافته های احتمالی این پژوهش چقدر برای شما جالب است.
- ✓ در صورت وجود مداخله مزایا و معایب آن در مقایسه با روش معمول ذکر شده است.

نکات مثبت:

مناسب است

نکات منفی:

به لحاظ هدف و ضرورت طرح:	خیلی خوب	خوب	کافی	متوسط	ناکافی

استراتژی و روش اجرا و قابل اجرا بودن در شرایط کشور

در نگارش نکات مثبت و منفی موارد زیر را در نظر بگیرید.

- ✓ شفافیت
- ✓ طراحی، روش اجرا و روش آنالیز به اندازه کافی تشریح شده است و براساس آن می توان مطالعه را تکرار کرد.
- ✓ چشم انداز رسیدن به اهداف با استفاده از روش اجرا پیشنهادی
- ✓ واقع گرایانه بودن حجم نمونه و امکانات در اختیار
- ✓ واقع گرایانه بودن توانایی ها، موانع و محدودیت های بالقوه ذکر شده

نکات مثبت:

روش کار به خوبی بیان شده است و مناسب است

نکات منفی:

به لحاظ روش اجرا طرح:	خیلی خوب	خوب	کافی	متوسط	ناکافی

پژوهشگران

در نگارش نکات مثبت و منفی موارد زیر را در نظر بگیرید.

- ✓ تخصص مناسب و چندوجهی بودن تیم پژوهشی
- ✓ سوابق و کارهای پژوهشی گذشته
- ✓ توان این تیم در به پایان رساندن موفق این پژوهش
- ✓ نیاز به متدولوژیست

نکات مثبت:

با توجه به مشارکت سایر مراکز بسیار مناسب است

نکات منفی:

ترکیب تیم پژوهشی	خیلی خوب	خوب	کافی	متوسط	ناکافی

فرم ارزیابی طرحهای پژوهشی

ارزیابی نوآوری، ارتباط با نیاز و اولویت جامعه و نحوه و زمان لازم برای بکارگیری دستاورد پژوهش در کشور

در نگارش نکات مثبت و منفی موارد زیر را در نظر بگیرید.

- ✓ این پژوهش چیزی را به دانسته های ما اضافه می کند.
- ✓ شکاف مهمی را در دانش پر یا در حوزه ای است که اختلاف نظر و نتایج متناقض وجود دارد.
- ✓ این پژوهش محصولی جدید شامل دارو، وسیله، روش درمانی/جراحی، دستورالعمل یا ... تولید می کند.
- ✓ تکرار پژوهش های قبلی یا در حال اجرا که شما مطلع هستید اما از روشی بهتر، دستگاهی دقیقتر برای اندازه گیری، حجم نمونه بالاتر برخوردار است یا انجام آن در جمعیت مورد نظر پژوهشگر (مثلا جمعیت ایرانی) لازم یا انتظار هست در این جمعیت نتایج متفاوت باشد.
- ✓ این پژوهش براساس نیاز و مشکل جامعه است.
- ✓ نفع پژوهش برای بیمار یا سلامت جامعه در کوتاه مدت و بلندمدت (کیفیت خدمات بهداشتی-درمانی، هزینه اثربخشی، کاهش افراد بیمار که کار یا مدرسه را ترک می کنند و ...) لحاظ شده است.
- ✓ امکان بکارگیری وسیع نتایج این پژوهش وجو دارد
- ✓ روش بکارگیری وسیع نتایج توضیح داده شده و قابل قبول است.

نکات مثبت:

پۆوهش بر اساس نیاز واقعی جامعه است و درجه نوآوری بالایی نیز دارد

نکات منفی:

دستاوردهای پژوهش در طرح تکمیل نشده است

نوآوری، ارتباط طرح با نیاز و اولویت جامعه ...	خیلی خوب	خوب	کافی	متوسط	ناکافی

بودجه

- ✓ هزینه مواد و وسایل و هزینه پرسنلی
- ✓ هزینه اثربخشی: بالانس بین توان، هزینه و منابعی که باید صرف شود و مزایا و نتایج احتمالی چگونه است.

نکات اصلاحی:

جمع کل هزینه ها در طرح ذکر نشده است

در مجموع بودجه درخواستی:	خیلی زیاد	واقع گرایانه	خیلی کم

اخلاق

در نگارش نکات اصلاحی و ارزیابی خود موارد زیر را در نظر بگیرید.

- ✓ با مطالعه دقیق چک لیست اخلاق (مطالعات انسانی و حیوانی) و در صورت وجود مداخله مزایا و معایب احتمالی آن را برای آزمودنی لحاظ نمایید.
- ✓ فرم رضایتنامه آگاهانه (در صورت نیاز) باید به زبان آزمودنی نوشته شود و اطلاعات کافی برای گرفتن تصمیم آگاهانه در آن وجود داشته باشد.
- ✓ هدف اصلی هر پژوهش‌باید ارتقای سلامت انسان‌ها توأم با رعایت کرامت و حقوق ایشان باشد.
- ✓ در پژوهش بر آزمودنی انسانی، سلامت و ایمنی آزمودنی‌ها در طول و بعد از اجرای پژوهش، بر تمامی مصالح دیگر اولویت دارد.
- ✓ پژوهش بر انسان فقط در صورتی توجیه‌پذیر است که منافع بالقوه‌ی آن برای هر فرد آزمودنی بیش‌تر از خطرهای آن باشد.
- ✓ مواردی از قبیل سرعت، سهولت کار، راحتی پژوهشگر، هزینه‌ی پایین‌تر و/ یا صرفاً عملی بودن آن به هیچ وجه نباید موجب قرار دادن آزمودنی در معرض خطر یا زیان افزوده یا تحمیل هر گونه محدودیت اختیار اضافی به وی شود.
- ✓ در کارآزمایی های بالینی دوسوکور که آزمودنی از ماهیت دارویی یا مداخله‌ای که برای وی تجویز شده بی‌اطلاع است، پژوهشگر باید تدابیر لازم جهت کمک‌رسانی به آزمودنی در صورت لزوم و در شرایط اضطراری را تدارک ببیند.
- ✓ در پژوهش‌های پزشکی که ممکن است به محیط زیست آسیب برسانند، باید احتیاط‌های لازم در جهت حفظ و نگهداری و عدم آسیب رسانی به محیط زیست انجام گیرد.
- ✓ در پژوهش‌هایی که از مواد بدنی (شامل بافت‌ها و مایعات بدن انسان) یا داده‌هایی استفاده می‌شود که هویت صاحبان آن‌ها معلوم یا قابل کشف و ردیابی است، باید برای جمع‌آوری، تحلیل، ذخیره‌سازی و /یا استفاده‌ی مجدد از آن‌ها رضایت آگاهانه گرفته شود.
- ✓ در مواردی که آگاه کردن آزمودنی درباره‌ی جنبه‌ای از پژوهش باعث کاهش اعتبار پژوهش می شود، ضرورت اطلاع‌رسانی ناکامل از طرف پژوهشگر باید توسط کمیته‌ی اخلاق تأیید شود. بعد از رفع عامل این محدودیت، باید اطلاع‌رسانی کامل به آزمودنی انجام گیرد.
- ✓ برخی از افراد یا گروه‌هایی از مردم، نظیر ناتوانان ذهنی، کودکان، جنین و نوزاد، بیماران اورژانسی، یا زندانیان، سرباز که ممکن است به عنوان آزمودنی در پژوهش شرکت کنند، نمی‌توانند برای دادن رضایت، آگاهی یا آزادی لازم را داشته باشند. این افراد یا گروه‌ها آسیب پذیر دانسته می‌شوند و باید مورد حفاظت ویژه قرار گیرند.
- ✓ پژوهشگر مسؤول رعایت اصل رازداری و حفظ اسرار آزمودنی‌ها و اتخاذ تدابیر مناسب برای جلوگیری از انتشار آن است. هم‌چنین، پژوهشگر موظف است که از رعایت حریم خصوصی آزمودنی‌ها در طول پژوهش اطمینان حاصل کند. هرگونه انتشار داده‌ها یا اطلاعات به دست آمده از بیماران باید بر اساس رضایت آگاهانه انجام گیرد.
- ✓ روش پژوهش نباید با ارزش‌های احتماعی، فرهنگی و دینی جامعه در تناقض باشد.
- ✓ مطالعات حیوانی باید با رعایت کامل اصول اخلاقی کار با حیوانات آزمایشگاهی انجام شوند.

نکات اصلاحی:

**به لحاظ اخلاقی:**	**غیرقابل اجرا**	**با دقت و نظارت زیاد**	**اخلاقی**	**غیرقابل ارزیابی**

نام و نام خانوادگی داور: داور اول

امضا
